# Cannabidiol Enhances Mitochondrial Metabolism and Antioxidant Defenses in Human Intestinal Epithelial Caco-2 Cells

**DOI:** 10.3390/nu16223843

**Published:** 2024-11-09

**Authors:** Alejandro Bravo Iniguez, Qi Sun, Qiaorong Cui, Min Du, Mei-Jun Zhu

**Affiliations:** 1 School of Food Science, Washington State University, Pullman, WA 99164, USA; a.bravoiniguez@wsu.edu (A.B.I.); qi.sun6@wsu.edu (Q.S.); qiaorong.cui@wsu.edu (Q.C.); 2 Department of Animal Sciences, Washington State University, Pullman, WA 99164, USA; min.du@wsu.edu

**Keywords:** AMPK, antioxidant activity, cannabidiol, intestinal epithelium, mitochondria

## Abstract

Background: The reintroduction of hemp production has resulted in increased consumption of cannabidiol (CBD) products, particularly CBD oil, yet their effects on intestinal health are not fully understood. Proper mitochondrial function and antioxidant defenses are vital for maintaining the intestinal epithelial barrier. AMP-activated protein kinase (AMPK) and peroxisome proliferator-activated receptor gamma coactivator (PGC)1α are key mediators of mitochondrial metabolism. Methods & Results: Using Caco-2 cells, we found that CBD oil promoted AMPK phosphorylation, upregulated differentiation markers, and enhanced PGC1α/SIRT3 mitochondrial signaling. CBD oil reduced reactive oxygen species production and increased antioxidant enzymes. Moreover, CBD oil also increased levels of citrate, malate, and succinate—key metabolites of the tricarboxylic acid cycle—alongside upregulation of pyruvate dehydrogenase and isocitrate dehydrogenase 1. Similarly, pure CBD induced metabolic and antioxidant signaling. Conclusions: CBD enhances mitochondrial metabolic activity and antioxidant defense in Caco-2 cells, making it a promising candidate for treating intestinal dysfunction.

## 1. Introduction

The intestinal epithelium is a self-renewing, tightly regulated barrier essential for nutrient absorption, tissue protection, and the coordination of immune responses [1]. Dysregulation of the epithelium, such as impaired barrier function, is characteristic of gut diseases such as inflammatory bowel disease (IBD) [2]. The intestinal epithelium is constantly renewed, depending on the proliferation and differentiation of stem cells residing in crypts. Extensive oxidative metabolic transition and mitochondrial biogenesis occur during epithelial differentiation [3]. Proper cellular energetics, orchestrated by functional mitochondria, are vital for epithelial health. Mice with enhanced intestinal oxidative phosphorylation and ATP production develop less severe colitis than their peers [4]. In humans, patients with colitis exhibit decreased levels of mitochondrial respiratory chain complexes compared to healthy counterparts [5].

Mitochondria are major producers of free radicals, and their dysfunction results in increased oxidative damage that contributes to the progression of pathological intestinal conditions [6]. The inability of the body to balance the production of free radicals, such as reactive oxygen species (ROS), with appropriate defense mechanisms results in damaging oxidative stress, which plays a role in the pathology of numerous conditions including IBD [7]. Managing ROS is an intricate process vital for maintaining redox homeostasis. The body employs various defenses to control levels of oxidative stress, including antioxidative enzymes, whose levels can be influenced by lifestyle factors and aging [8]. Patients with gastrointestinal diseases exhibit both elevated levels of ROS and decreased activity of antioxidative enzymes [9]. Inhibition of these mediators increases ROS levels and promotes inflammasome activation [10]. Thus, it is important to identify strategies to maintain adequate antioxidative activity.

NF-E2-related factor 2 (NRF2) is an important transcription factor that regulates the expression of antioxidant genes and controls ROS levels in the intestines [11]. NRF2 is regulated by Kelch-like ECH-associated protein 1 (KEAP1), and it binds to the antioxidant response element (ARE) to promote the expression of enzymes such as NAD(P)H quinone dehydrogenase 1 (NQO1) and heme oxygenase 1 (HO-1) [12]. In the canonical pathway of NRF2 activation, oxidative stress decreases the ability of KEAP1 to sequester NRF2, allowing NRF2 to translocate to the nucleus and activate ARE-driven gene expression [13]. Alternatively, the scaffold protein p62 plays a role in the noncanonical activation of NRF2 by disrupting the interaction between KEAP1 and NRF2 [14,15]. Several upstream pathways regulate NRF2/HO-1, including phosphoinositide 3-kinase (PI3K)/AKT [16], p38 mitogen-activated protein kinase (MAPK) [17], and AMP-activated protein kinase (AMPK) [18]. AMPK also regulates intestinal differentiation and barrier function through the activation of caudal type homeobox 2 (CDX2) [19], illustrating the interconnection between metabolic and antioxidant pathways, which is essential for maintaining intestinal homeostasis.

Cannabidiol (CBD) is a non-psychoactive cannabinoid found in cannabis that can reach the gut depending on the method of delivery [20,21]. In the human colorectal carcinoma cell line Caco-2, CBD reduces ROS production and improves transepithelial resistance upon exposure to H_2_O_2_ or H_2_O_2_/Fe^2+^ [22,23]. We previously demonstrated that mice with dextran sodium sulfate (DSS)-induced colitis that received 200 mg kg^−1^ CBD in their diet for 5 weeks showed reduced inflammation, associated with increased phosphorylation and activation of AMPK [24].

Several studies showed the effects of CBD in improving mitochondrial energetics. Hippocampal neurons treated with 5 µM CBD exhibit greater mitochondrial respiration compared to untreated neurons [25]. In mice, intraperitoneal injections of CBD ameliorate the negative effects of doxorubicin on mitochondrial biogenesis in the myocardium [26]. The effect of CBD on intestinal mitochondrial energetics and signaling remains to be elucidated. This study used human epithelial Caco-2 cells to investigate the effects of CBD on the antioxidant and metabolic activities of colon cells. We hypothesized that CBD treatment upregulates the phosphorylation of AMPK in Caco-2 cells, which is accompanied by enhanced mitochondrial energetics and the upregulation of antioxidant enzymes.

## 2. Materials and Methods

### 2.1. Cannabidiol

A commercial full-spectrum CBD hemp oil was purchased directly from the manufacturer (Nutra Pure LLC, Vancouver, WA, USA). As per the test results provided by the producer, analysis by a published method [27] determined the CBD content in the oil to be 18.3 mg/mL, while other cannabinoids were below the limit of quantification. A CBD stock solution (10 mM) was prepared by diluting the oil in dimethylsulfoxide (DMSO) (VWR, Radnor, PA, USA). Additionally, cannabidiol (≥98%) was purchased from Cayman Chemical (Ann Arbor, MI, USA) and dissolved in DMSO to prepare a stock solution at 10 mM. All CBD stock solutions were stored at −20 °C.

### 2.2. Cell Line

Caco-2 cells were obtained from the American Type Culture Collection (Manassas, VA, USA). The cells were routinely cultured in Dulbecco’s modified Eagle medium (DMEM) (Sigma; St. Louis, MO, USA) supplemented with 10% fetal bovine serum (Sigma), 100 units/mL of penicillin G, and 100 µg/mL of streptomycin (Sigma) at 37 °C with 5% CO_2_. Cells were seeded into 12-well plates for analyses unless stated otherwise. Following overnight incubation, cells were treated with or without 10µM CBD for 1, 2, or 4 days.

### 2.3. Intracellular Reactive Oxygen Species (ROS) Measurement

Levels of intracellular ROS were evaluated as previously described [28]. In brief, Caco-2 cells were seeded into 96-well plates and cultured in complete DMEM overnight, followed by incubation with media containing the cell-permeable fluorescent probe, 2,7-dichlorofluorescein diacetate (H_2_DCFDA) (MilliporeSigma, Burlington, MA, USA), for 45 min. After replacing the media, the cells were treated with or without CBD and with or without H_2_O_2_ for 24 h. Fluorescence was measured using a BioTek Synergy H1 microplate reader (Agilent Technologies, Palo Alto, CA, USA) with an excitation wavelength of 485 nm and an emission wavelength of 530 nm. The fluorescence of H_2_DCFDAobtained from the plate reader was reported in arbitrary units and normalized to the values of the untreated controls.

### 2.4. Immunoblotting

Proteins were extracted from Caco-2 cells, separated by SDS-PAGE gels, and transferred onto nitrocellulose membrane as described previously [29,30]. Antibodies against acetyl-CoA carboxylase (ACC), AMPK, CDX2, catalase, HO-1, isocitrate dehydrogenase 1 (IDH1), p62, p-ACC, p-AMPK, pyruvate dehydrogenase (PDH), sirtuin3 (SIRT3), and superoxide dismutase (SOD)2 were purchased from Cell Signaling Technology (Danvers, MA, USA). The antibody for claudin-2 was purchased from Thermo Fisher Scientific (Waltham, MA, USA), while the antibody for proliferator-activated receptor gamma coactivator 1 alpha (PGC1α) was obtained from ProteinTech (Rosemont, IL, USA). The antibody for SOD1 was purchased from Santa Cruz Biotechnology (Dallas, TX, USA). Horseradish peroxidase-coupled anti-rabbit or anti-mouse IgGs were used for visualization by chemiluminescence. Band density quantification was normalized to the signal of β-tubulin. Data are presented as relative to the control group.

### 2.5. Quantitative Reverse Transcription PCR (qRT-PCR) Analysis

Total mRNA was extracted from Caco-2 cells or animal tissue using Trizol Reagent (Thermo Fisher) per the manufacturer’s instructions. Reverse transcription was completed using an iScript™ kit (Bio-Rad, Hercules, CA, USA). The produced cDNA served as templates for qRT-PCR analysis, using SYBR Green Master Mix (Bio-Rad) and a CFX96™ Real-Time PCR Detection System (Bio-Rad). The primers used for qRT-PCR are listed in Supplementary Materials Table S1, with 18S serving as the housekeeping gene.

### 2.6. Tricarboxylic Acid Cycle Metabolite Analysis by GC-MS

Caco-2 cells were treated with or without CBD for 2 days. Following the treatment period, the cells were collected and processed for analysis using an Agilent 7890B gas chromatography system equipped with a 5977A single-quadrupole mass spectrometer and a 7693 autosampler system (Agilent Technologies), following previously established procedures [31]. The column utilized was an HP-5 ms column (30 m × 250 µM i.d., 0.25 µM film thickness; Agilent Technologies). Ribitol, purchased from Sigma, served as the internal standard. Citrate and malate standards were purchased from Sigma. The succinate standard was purchased from ThermoFisher Scientific. The relative abundances of metabolites were determined by calculating the area ratios of the target peaks to the ribitol (internal standard) peaks.

### 2.7. Statistical Analysis

Statistical analysis was performed as previously described using GraphPad Prism 7 [31]. The data are presented relative to the control group as mean ± SEM (standard error of the mean). Treatments were compared using either a two-tailed Student’s *t*-test or one-way ANOVA. Significance was determined using a *p*-value ≤ 0.05.

## 3. Results

### 3.1. CBD Oil Induces Phosphorylation of AMPK and Upregulates Downstream Targets

**Figure 1:**
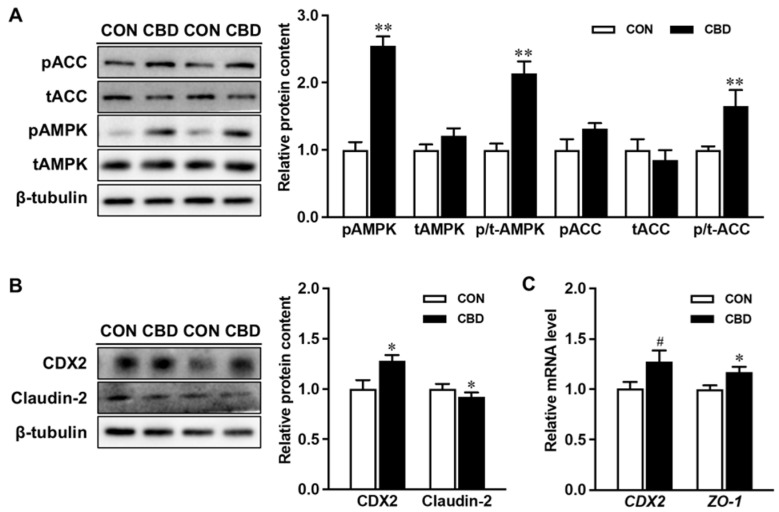
Cannabidiol oil induces AMP-activated protein kinase (AMPK) phosphorylation and upregulates downstream targets overseeing epithelial differentiation and barrier function. (**A**) Protein contents of p-AMPK, t-AMPK, p- acetyl-CoA carboxylase (ACC), and t-ACC. (**B**) The protein content of caudal type homeobox 2 (CDX2) and claudin-2. (**C**) mRNA expression of *CDX2* and zonula occludens-1 (*ZO-1*). CON: untreated Caco-2 cells; CBD: Caco-2 cells treated with 10 µM cannabidiol (CBD) oil. Mean ± SEM, *n* = 4, #: *p* ≤ 0.10; *: *p* ≤ 0.05; **: *p* ≤ 0.01.

Incubation with CBD oil increased the phosphorylation of AMPK compared to controls (Figure 1A). CBD oil treatment also enhanced the phosphorylation of ACC, a downstream target of AMPK (Figure 1A). Moreover, at both the protein and mRNA levels, CBD oil exhibited a promotive effect on CDX2, another downstream target of AMPK and the key transcription factor governing epithelial differentiation (Figure 1B,C). Additionally, exposure to CBD oil decreased the protein level of claudin-2 and increased the mRNA level of zonula occludens-1 (*ZO-1*), indicating positive effects on epithelial differentiation and barrier function (Figure 1B,C).

### 3.2. CBD Oil Treatment Promotes Mitochondrial Energetics

**Figure 2:**
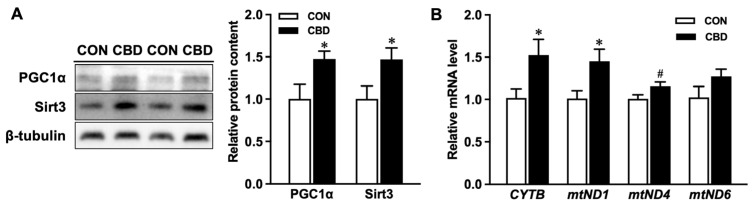
Cannabidiol oil enhances mitochondrial signaling and activity. (**A**) Protein contents of Peroxisome proliferator-activated receptor gamma coactivator 1 alpha (PGC1α) and sirtuin 3 (SIRT3). (**B**) mRNA expression of cytochrome b (*CYTB*), mitochondrially encoded NADH-ubiquinone oxidoreductase core subunit 1 (*mtND1)*, *mtND4*, and *mtND6*. CON: untreated Caco-2 cells; CBD: Caco-2 cells treated with 10 µM cannabidiol (CBD) oil. Mean ± SEM, *n* = 4, #: *p* ≤ 0.10; *: *p* ≤ 0.05.

**Figure 3:**
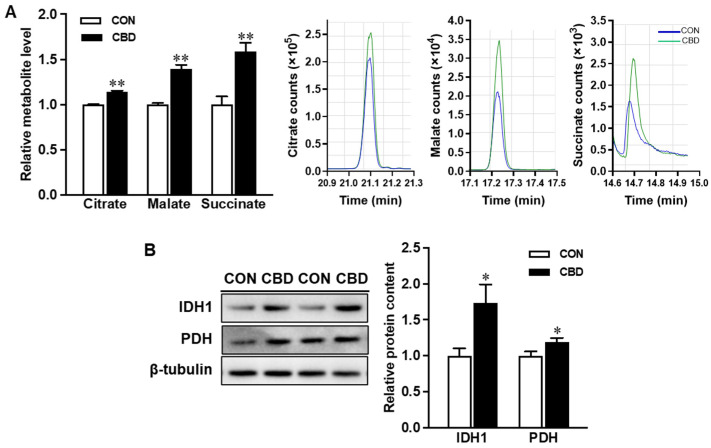
Cannabidiol oil alters tricarboxylic acid (TCA) cycle activity and metabolite levels. (**A**) Levels of TCA cycle metabolites obtained from gas chromatography/mass spectrometry and representative peaks for citrate, malate, and succinate. (**B**) Protein contents of isocitrate dehydrogenase 1 (IDH1) and pyruvate dehydrogenase (PDH). CON: untreated Caco-2 cells; CBD: Caco-2 cells treated with 10 µM cannabidiol (CBD) oil. Mean ± SEM, *n* = 3–4, *: *p* ≤ 0.05; **: *p* ≤ 0.01.

CBD oil treatment increased protein contents of the transcription factor Peroxisome proliferator-activated receptor gamma coactivator 1 alpha (PGC1α) and the deacetylase enzyme, Sirtuin 3 (SIRT3) (Figure 2A). Treatment with CBD oil also upregulated the mRNA expression of the mitochondrially encoded NADH-ubiquinone oxidoreductase core subunit 1 (*mtND1*), *mtND4*, and cytochrome b (*CYTB*) (Figure 2B). Additionally, CBD oil promoted oxidative phosphorylation in Caco-2 cells. GC-MS analysis of key tricarboxylic acid (TCA) cycle metabolites revealed elevated contents of citrate, malate, and succinate in CBD-treated cells (Figure 3A). This effect was accompanied by increased protein contents of isocitrate dehydrogenase 1 (IDH1), an enzyme in the TCA cycle responsible for the conversion of isocitrate to alpha-ketoglutarate (αKG), and pyruvate dehydrogenase (PDH), the enzyme responsible for directing pyruvate to oxidative phosphorylation (Figure 3B).

### 3.3. CBD Oil Suppresses ROS Formation and Upregulates Antioxidants

**Figure 4:**
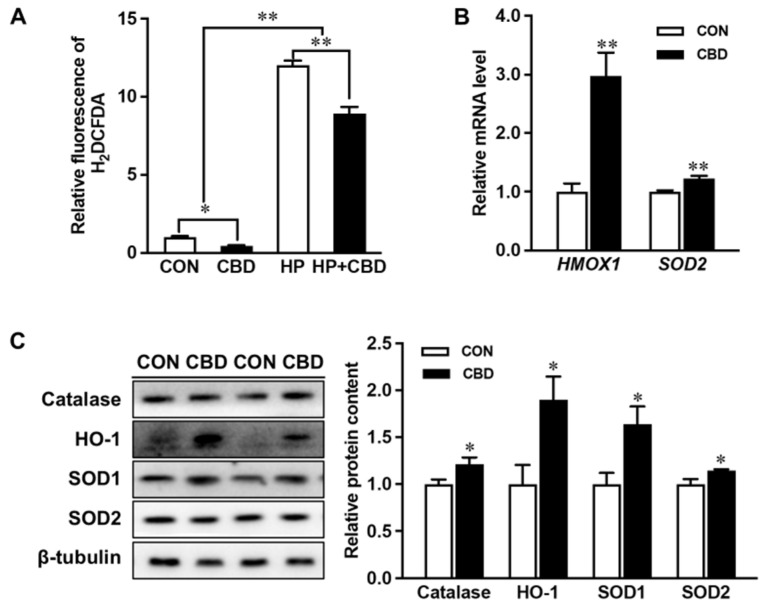
Cannabidiol oil mitigates reactive oxygen species (ROS) production and enhances antioxidant expression. (**A**) ROS production measured with H2DCFDA. (**B**) mRNA expression of heme oxygenase 1 gene (*HMOX1)* and superoxide dismutase 2 (*SOD2)*. (**C**) Protein contents of catalase, heme oxygenase 1 (HO-1), SOD1, and SOD2. CON: untreated Caco-2 cells; CBD: Caco-2 cells treated with 10 µM cannabidiol (CBD) oil. Mean ± SEM, *n* = 4, *: *p* ≤ 0.05; **: *p* ≤ 0.01.

Treating Caco-2 cells with 10 µM CBD oil lowered levels of ROS with or without the H_2_O_2_ challenge (Figure 4A). CBD oil also induced the expression of the antioxidant enzyme, HO-1 (Figure 4B). Moreover, the protein contents of catalase SOD1, and to a lesser extent SOD2, were increased (Figure 4B). At the mRNA level, CBD oil upregulated heme oxygenase 1 gene (*HMOX1)* and *SOD2* expression (Figure 4C).

### 3.4. Exposure to CBD Oil Upregulates NRF2 Signaling Pathway

**Figure 5:**
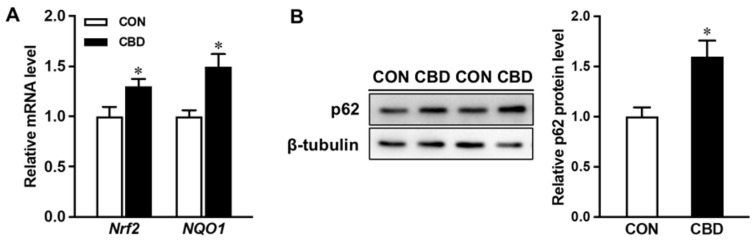
Cannabidiol oil targets NF-E2-related factor 2 (NRF2) signaling. (**A**) mRNA expression of *Nrf2* and NAD(P)H quinone dehydrogenase 1 (*NQO1)*. (**B**) Protein content of p62. CON: untreated Caco-2 cells; CBD: Caco-2 cells treated with 10 µM cannabidiol (CBD) oil. Mean ± SEM, *n* = 4, *: *p* ≤ 0.05.

Consistent with the upregulation of HO-1 protein and mRNA (*HMOX1)* levels in CBD oil-treated cells, the mRNA expression of *NRF2* was also elevated (Figure 5A). Accordingly, the mRNA level of *NQO1*, a target gene of NRF2 encoding the detoxification enzyme, was increased in CBD oil-treated cells. The content of p62 was also upregulated in CBD oil-treated cells (Figure 5B).

### 3.5. Pure CBD Promotes Signaling Pathways Similarly to CBD Oil

**Figure 6:**
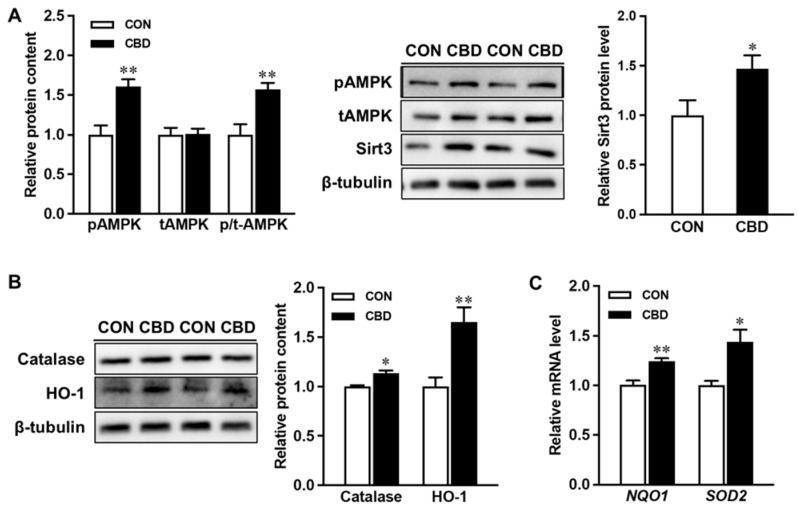
Pure cannabidiol induces phosphorylation of AMPK and upregulates both mitochondrial and antioxidant signaling. (**A**) Protein contents of p- AMP-activated protein kinase (AMPK), t-AMPK, and sirtuin3 (SIRT3). (**B**) Protein contents of catalase and heme oxygenase 1 (HO-1). (**C**) mRNA expression of NAD(P)H quinone dehydrogenase 1 *(NQO1)* and superoxide dismutase 2 (*SOD2)*. CON: untreated Caco-2 cells; CBD: Caco-2 cells treated with 10 µM cannabidiol (CBD). Mean ± SEM, *n* = 4, *: *p* ≤ 0.05; **: *p* ≤ 0.01.

While CBD is the major constituent in commercial CBD oils, it is common for them to contain other cannabinoids at much smaller levels. To confirm that CBD exerts positive effects on AMPK and related signaling, Caco-2 cells were further treated with 10 µM pure CBD compound. Consistently, pure CBD increased phosphorylation of AMPK and upregulated SIRT3 (Figure 6A). Pure CBD exerted beneficial effects on antioxidant signaling, elevating protein levels of HO-1 and catalase (Figure 6B), as well as the mRNA expression of *NQO1* and *SOD2* (Figure 6C). Together these findings show that CBD targets mitochondrial and antioxidant signaling to improve intestinal epithelial health.

## 4. Discussion

The average daily intake of CBD among adults who regularly consume CBD-containing products is 50.3 ± 40.7 mg [32]. CBD exhibits poor bioavailability, hampered by its low water solubility [33], and approximately one-third of orally ingested CBD is excreted in the feces [34]. Pharmacokinetic studies with humans have largely focused on serum levels following oral intake. After ingesting an oral capsule containing 10 mg CBD, the maximum concentration measured in serum was 2.47 ng/mL (7.85 nM) [35]. In a different study, a higher oral dose of 200 mg CBD resulted in a maximum serum concentration of 148 ng/mL (470.63 nM) [36]. Multiple factors affect the oral absorption of CBD, including the presence of lipids, which enhances CBD absorption [35,37]. Following oral delivery in rats, CBD levels detected in intestinal lymph far exceeded serum levels, being 250-fold greater [37], suggesting the importance of understanding its effects on gut epithelial health. If the same holds following oral intake in humans, micromolar levels of CBD may be obtainable in the intestines.

Among CBD product consumers, drops are the most commonly used type of product [38]. In this experiment, we employed commercially available CBD oil to best reflect exposure to the average consumers. This CBD oil utilized hempseed oil as a carrier, which contains essential fatty acids and bioactive tocopherols [39]. To account for this, we also utilized pure CBD in the present study. Given that concentration and dosage instructions differ between CBD products, the use of a single treatment condition is a limitation of the present study.

The present study employed the Caco-2 cell line to model the intestinal epithelium of the colon. The cultured cells mimic epithelial differentiation upon achieving confluence which dictated our choice of treatment times [19]. In Caco-2 cell monolayers, 10 µM CBD treatment potentiated the recovery of transepithelial resistance following a challenge with ethylenediaminetetraacetic acid [40]. CBD treatment also resulted in elevated mRNA expression of *ZO-1* [40]. In the present study, CBD oil treatment similarly increased the mRNA expression of *ZO-1*. In agreement with our previous observations in mice with DSS-induced colitis, CBD oil and pure CBD increased the phosphorylation of AMPK in Caco-2 cells. AMPK, a regulator of energy homeostasis, plays a key role in the homeostasis of the intestinal epithelium [19,41]. Considering that AMPK activation upregulates differentiation transcription factor CDX2 at both the mRNA and protein levels [42], the ability of both CBD to activate AMPK and upregulate its downstream targets holds promise as a means to strengthen intestinal epithelial barrier function.

Proper differentiation relies on mitochondrial function, characterized by increased oxidative phosphorylation and mitochondrial biogenesis as epithelial cells differentiate and migrate away from the crypts [43,44]. In intestinal epithelial cells, the proinflammatory signal, tumor necrosis factor-alpha (TNF-α) induces mitochondrial dysfunction, reducing oxygen consumption and mitochondrial membrane potential [45]. It also decreases the expression of alkaline phosphatase, a marker for intestinal epithelial differentiation [46]. CBD oil treatment upregulated the protein contents of PGC1α and SIRT3, a deacetylase controlling mitochondrial quantity, metabolism, and antioxidant activity [47,48].

Furthermore, CBD oil upregulated the TCA cycle enzyme IDH1, responsible for synthesizing α-ketoglutarate, a vital cofactor for epigenetic modifications that is required for the proper differentiation of epithelial cells [49,50]. PDH was also increased in CBD-treated cells. Impairment of the PDH in cancer cells favors greater glycolysis at the expense of mitochondrial oxidation [51]. Changes to TCA cycle enzyme protein levels were accompanied by changes in metabolite levels. CBD treatment increased the intracellular content of citrate, malate, and succinate in Caco-2 cells. In pigs, succinate increased epithelial tight junction protein content [52]. Together, our findings suggest that CBD enhances mitochondrial metabolic function in colon epithelial cells, which may contribute to its beneficial effects.

CBD oil treatment ameliorated ROS production in Caco-2 cells both in the presence or absence of H_2_O_2_. This effect was accompanied by the upregulation of antioxidant enzymes including HO-1. Similar observations of NRF2 and HO-1 induction have been reported in keratinocytes [53] and endothelial cells [54] treated with 1 and 6 µM CBD, respectively. The scaffold protein p62 can facilitate the degradation of KEAP1, impeding its interactions with NRF2 [55], and also participates in a positive feedback loop with NRF2 [56]. We observed the upregulation of p62 in CBD oil-treated Caco-2 cells. Given its role in regulating the expression of important antioxidants and detoxification enzymes, proper NRF2 expression is crucial for intestinal health. Mice deficient in NRF2 showed increased susceptibility to colitis induced by DSS [57]. Conversely, the activation of NRF2 and the upregulation of its antioxidant signaling pathways in mice mitigated DSS-induced disease severity [58]. In DSS-induced colitis, HO-1 is negatively regulated by transcription factor BTB domain and CNC homology 1 (BACH1), and mice deficient in BACH1 display elevated levels of colonic HO-1 and decreased disease activity [59]. Co-treatment with ZnPP, an inhibitor of HO-1, negated the beneficial effects of BACH1 deficiency on disease activity [59]. Promoting proper expression of HO-1 and other antioxidant defenses through phytochemicals such as CBD may serve as an approach for combatting intestinal dysfunction.

## 5. Conclusions

As the burden generated by IBD persists, multiple prevention and treatment strategies are needed. The beneficial effects of plant-derived bioactive compounds, such as resveratrol, which ameliorate disease activity of IBD in experimental animal models of colitis, are partially through the promotion of antioxidant activity [60]. Our findings showcase the ability of CBD to combat oxidative stress in colonic epithelial cells, shedding light on potential mechanisms behind these effects such as induction of NRF2/HO-1 signaling. Additionally, our study reveals that CBD treatment impacts mitochondrial energetics and related signaling, inducing the phosphorylation of AMPK and the upregulation of PGC1α and SIRT3. Given that the maintenance of the intestinal epithelium is an energy-demanding process reliant on intricate regulation of mitochondrial activity, our findings help understand the potential of CBD to safeguard against intestinal epithelial dysregulation.

## Data Availability

The data presented in this study are available upon request.

## Supplementary Materials

The following supporting information can be downloaded at: https://www.mdpi.com/article/10.3390/nu16223843/s1, Table S1: Lists the primer sets used for quantitative RT-qPCR. References [61,62,63] are cited in Supplementary Materials.
