# Supplementary material for: Cannabidiol Enhances Mitochondrial Metabolism and Antioxidant Defenses in Human Intestinal Epithelial Caco-2 Cells
Source: Nutrients. 2024 Nov 9;16(22):3843. doi: 10.3390/nu16223843 (PMC11597683; doi:10.3390/nu16223843)
Supplement: Supplementary file 1 [file nutrients-16-03843-s001.zip › nutrients-3302740-supplementary.pdf]

nutrients MDPI

Table S1. Primer sets used for quantitative RT-PCR in the study.

Gene Name	Accession No.	Product Size	Direction	Sequence (5'→3')	Source
CDX2	NM_007673.3	92bp	Forward	TTCTGGACAAGGACGTGAGC	[42]
			Reverse	CTGCGGAGGACTGACAAAGT	
HMOX1	NM_002133.3	247bp	Forward	AAGACTGCGTTCCTGCTCAAC	[61]
			Reverse	AAAGCCCTACAGCAACTGTCG	
CYTB	JN034136.1	248bp	Forward	TGGCTGAATCATCCGCTACC	[31]
			Reverse	TCCCAATGTATGGGATGGCG	
mtND1	NC_012920.1	235bp	Forward	TCCTGCCATCATGACCCTTG	[62]
			Reverse	TCAGGGGAGAGTGCGTCATA	
mtND4	NC_012920.1	278bp	Forward	CTCGCTAACCTCGCCTTACC	[62]
			Reverse	CCGGTAATGATGTCGGGGTT	
mtND6	NC_012920.1	221bp	Forward	CACCCACAGCACCAATCCTA	[62]
			Reverse	TTGTTAGCGGTGTGGTCGG	
NFE2L2	NM_006164.4	129bp	Forward	CAACTACTCCCAGGTTGCCC	[63]
			Reverse	AGTGACTGAAACGTAGCCGA	
NQO1	NM_001286137.1	134bp	Forward	CCTTGTGATATTCCAGAGTGGC	This study
			Reverse	CCAGGCGTTTCTTCCATCCT	
SOD2	NM_000636.3	249bp	Forward	CACTAGCAGCATGTTGAGCC	[31]
			Reverse	GCTGTAACATCTCCCTTGGC	
ZO-1	NM_001301025.3	141bp	Forward	CTCACCACAAGCGCAGCCAC	This study
			Reverse	ACAGCAGAGGTTGATGATGC	
18S	NR_146119.1	76bp	Forward	CGCACGGCCGGTACAGTGAA	[31]
			Reverse	GGGAGAGGAGCGAGCGACCA	
